# Sociodemographic, clinical and laboratory factors on admission associated with COVID-19 mortality in hospitalized patients: A retrospective observational study

**DOI:** 10.1371/journal.pone.0235107

**Published:** 2020-06-25

**Authors:** Mario Rivera-Izquierdo, María del Carmen Valero-Ubierna, Juan Luis R-delAmo, Miguel Ángel Fernández-García, Silvia Martínez-Diz, Arezu Tahery-Mahmoud, Marta Rodríguez-Camacho, Ana Belén Gámiz-Molina, Nicolás Barba-Gyengo, Pablo Gámez-Baeza, Celia Cabrero-Rodríguez, Pedro Antonio Guirado-Ruiz, Divina Tatiana Martín-Romero, Antonio Jesús Láinez-Ramos-Bossini, María Rosa Sánchez-Pérez, José Mancera-Romero, Miguel García-Martín, Luis Miguel Martín-delosReyes, Virginia Martínez-Ruiz, Pablo Lardelli-Claret, Eladio Jiménez-Mejías

**Affiliations:** 1 Preventive Medicine and Public Health Service, Hospital Universitario Clínico San Cecilio, Granada, Spain; 2 Department of Preventive Medicine and Public Health, University of Granada, Granada, Spain; 3 Instituto Biosanitario de Granada, IBS.Granada, Granada, Spain; 4 Neurology Service, Hospital Universitario Clínico San Cecilio, Granada, Spain; 5 Psychiatry Service, Hospital Universitario Clínico San Cecilio, Granada, Spain; 6 Pulmonology Service, Hospital Universitario Clínico San Cecilio, Granada, Spain; 7 Clinical Medicine and Public Heath, Doctorate Program, University of Granada, Granada, Spain; 8 SEMERGEN-UGR Chair of Teaching and Research in Family Medicine, University of Granada, Granada, Spain; 9 CIBER de Epidemiología y Salud Pública de España, CIBERESP, Madrid, Spain; BronxCare Health System, Affiliated with Icahn School of Medicine at Mount Sinai, UNITED STATES

## Abstract

**Background:**

To identify and quantify associations between baseline characteristics on hospital admission and mortality in patients with COVID-19 at a tertiary hospital in Spain.

**Methods and findings:**

This retrospective case series included 238 patients hospitalized for COVID-19 at Hospital Universitario Clínico San Cecilio (Granada, Spain) who were discharged or who died. Electronic medical records were reviewed to obtain information on sex, age, personal antecedents, clinical features, findings on physical examination, and laboratory results for each patient. Associations between mortality and baseline characteristics were estimated as hazard ratios (HR) calculated with Cox regression models.

Series mortality was 25.6%. Among patients with dependence for basic activities of daily living, 78.7% died, and among patients residing in retirement homes, 80.8% died. The variables most clearly associated with a greater hazard of death were age (3% HR increase per 1-year increase in age; 95%CI 1–6), diabetes mellitus (HR 2.42, 95%CI 1.43–4.09), SatO_2_/FiO_2_ ratio (43% HR reduction per 1-point increase; 95%CI 23–57), SOFA score (19% HR increase per 1-point increase, 95%CI 5–34) and CURB-65 score (76% HR increase per 1-point increase, 95%CI 23–143).

**Conclusions:**

The patients residing in retirement homes showed great vulnerability. The main baseline factors that were independently associated with mortality in patients hospitalized for COVID-19 were older age, diabetes mellitus, low SatO_2_/FiO_2_ ratio, and high SOFA and CURB-65 scores.

## Introduction

Since 11 March 2020, when the World Health Organization (WHO) declared a worldwide SARS-CoV-2 pandemic, there have been more than 213,000 confirmed cases in Spain (as of 25 April 2020) and 22,157 deaths, representing a mortality rate of 47 per 100,000 inhabitants [1]. Different reports on the status of the COVID-19 epidemic published in Spain by the Ministry of Health indicate that this disease is slightly more prevalent in women, in people older than 50 years, and in people with two or more previous illnesses, particularly hypertension (HT) (47.4%), cardiovascular disease (CVD) (32.3%), and diabetes mellitus (DM) (16.1%) [2,3].

Among confirmed cases of infection, 48.7% patients have required hospitalization mainly for pneumonia and adult respiratory distress syndrome (ARDS). Among patients with pneumonia, men, people older than 60 years, and people with CVD and/or DM are overrepresented [4,5]. Among Spanish patients the most frequent symptoms have been fever (75.7%), cough (75.5%) and dyspnoea (48.3%) [6]. In 5% of hospitalized patients, the clinical course progressed to critical status requiring admission to the intensive care unit, and 2.3% have died [7,8].

Radiographic findings in patients with pneumonia are highly variable depending on–among other factors–the time elapsed since symptom onset [9,10]. However, the most frequent findings are bilateral infiltrates with an interstitial or ground glass pattern, or bilateral alveolar opacities [11,12]. Alterations in laboratory values are also frequent and varied, depending on disease severity, e.g. anaemia, leucocytosis, neutrophilia, lymphopenia, and elevated creatinine, erythrocyte sedimentation velocity (ESV), C-reactive protein (CRP), procalcitonin, lactate dehydrogenase (LDH), serum ferritin, troponins, and cytokines (among other markers) [12,13]. The most frequent laboratory findings in seriously ill patients are lymphopenia, hypoalbuminemia, and increased ferritin and D-dimer; most of these alterations probably reflect increases in cytokines levels or the “cytokine storm” phenomenon (IL-2R, IL-6, IL-10 and TNFα) [14,15].

Few studies to date have shed light on the main factors associated with fatality rates in hospitalized patients. Most notably, a retrospective cohort study of 191 patients at two hospitals in China reported 41 deaths, and analysed the association of sex, age, chronic obstructive lung disease, coronary disease, diabetes, hypertension, chronic kidney disease, and other comorbidities on the risk of death due to COVID-19. According to the multivariate models in this study, associations were detected only for patient age and two parameters measured on admission: Sequential Organ Failure Assessment (SOFA) score and D-dimer concentration greater than 1 μg/ml [16]. Other studies in similar conditions were made for Middle East respiratory syndrome (MERS) [17] and severe acute respiratory syndrome (SARS) [18] but the number of studies analysing prognosis factors on COVID-19 remain insufficient to date.

The aim of this study was to identify and quantify the associations between baseline characteristics on hospital admission in patients with COVID-19 infection (e.g. personal antecedents, physical examination and clinical findings, and laboratory results) and mortality at a tertiary hospital in Spain.

## Materials and methods

This retrospective case series comprised 238 patients admitted for COVID-19 to Hospital Universitario Clínico San Cecilio, and who died or were discharged, between 16 March and 10 April 2020. This 480-bed tertiary hospital is located in the city of Granada (southern Spain), and provides specialized health care services to a total population of 291,797 inhabitants. Only positive PCR test was considered as diagnosis criteria for the patients included in our series. Therefore, patients presenting compatible symptoms and negative test were not included in the present study. The information detailed below was collected from electronic medical records for each patient.

### General information on admission and recorded in the emergency service report

Sex, age (analysed as a continuous variable and also stratified into groups of 30–39, 40–49, 50–59, 60–69, 70–79, and 80 years or older), presence of comorbidities (HT; DM; previous lung disease; chronic kidney failure; CVD, or active neoplasia), dependence for basic activities of daily living (BADL) (yes or no), clinical findings on admission (low-grade or higher fever, dry cough, productive cough, haemoptysis, dyspnoea, anosmia, ageusia, headache, nausea, diarrhoea, other gastrointestinal symptoms, myalgia, fatigue, tiredness, general malaise, exanthema, odynophagia), smoking status (never smoker, current smoker, or ex-smoker) and place of residence (own home or retirement facility).

### Variables recorded from physical examination and laboratory tests on admission

Systolic and diastolic blood pressure, heart rate, temperature, basal oxygen saturation (SatO_2_), externally supplied oxygen (litres), fraction of inspired oxygen (FiO_2_), SatO_2_/FiO_2_ ratio, haemoglobin, lymphocyte count, neutrophil count, platelet count, D-dimer level, glycaemia, total bilirubin, LDH, ferritin, CRP, procalcitonin, troponins, creatinine, kidney function (elevated urea and creatinine), partial O_2_ and CO_2_ pressure in arterial blood (PO_2_ and PCO_2_), CO_2_, arterial blood pH, SOFA score, CURB-65 score, and X-ray findings on admission (categorized as dichotomous variables), e.g. diffuse opacities, interstitial opacities, hilar congestion or pleural effusion.

### Analysis of outcome variables: Death and days of hospital stay

Central tendency and dispersion were estimated for quantitative variables, and frequency distribution was determined for categorical variables. For each category in the latter, we estimated the distribution of discharges and deaths. Because our case series was defined on the basis of outcomes (discharge or death), it cannot be considered a true cohort of admitted patients, given that by definition we excluded patients who, at the end of the recruitment period (10 April 2020), remained hospitalized (censured data). For these patients the mean duration of hospital stay can be assumed to be longer than for patients included in the present case series. Because the length of hospital stay tends to be inversely associated with mortality, this association would lead to selection bias, which in turn would result in overestimating the proportion of deaths in our sample. In addition, it could potentially skew (in either direction) the magnitude of the associations between patient characteristics on admission and death. To compensate for this source of bias we opted to use time to death as a dependent variable and used Cox proportional hazards regression models to calculate hazard ratios (HR) in order to quantify the magnitude of associations between hazard or instantaneous death rate and patients’ baseline characteristics.

Because of the low number of patients compared to the number of independent variables considered in this analysis, we used a three-step modelling process. First, we developed separate models for each baseline variable and for age. Next we defined subgroups of baseline variables (sociodemographic, symptoms, comorbidities, physical examination on admission, laboratory values, and X-ray findings), and used a stepwise process to build multivariate models for each variable (including age in all models). The variables selected in each model where then used in a stepwise procedure to build a final model that included baseline variables that were retained. In all cases the P value for inclusion in the model was set at 0.05, and the P value for exclusion was set at 0.10. The 95%CI was calculated for each HR. All data were analysed with STATA software (version 15.0) [19].

### Ethics

This study met the requirements for appropriateness to achieve the intended aims and complied with all ethical principles pertinent to this type of study design. All data were fully anonymized before the analyses. No informed consent was required due to the characteristics of the design. The study was approved by the Research Ethics Committee of Granada province (CEI) on 13 April 2020.

## Results

Slightly more than half of our patients were males (55.0%, n = 131). The age range was 24 to 97 years with a mean of 64.7, standard deviation 15.4, and median 67 years. Most patients (60.5%) were 60 years old or more, and the largest age stratum was 70 to 79 years. At least one comorbidity was present on admission in 56.7% of the sample. Among the most prevalent diseases were HT (48.7%), DM (21.9%), and CVD (22.7%). About one fifth of the patients (19.8%) had dependence for BADL, and 10.9% lived in retirement homes (Table 1). On admission, the most frequent clinical profile was characterized by low-grade fever (89.5%), dry cough (80.7%), general malaise (63.5%), dyspnoea (61.3%) and tiredness (59.2%) (Table 1).

**Table 1:** Sociodemographic and clinical characteristics, and distribution of deaths in each category.

Groups	Category		N	%	Deaths	
					N	%^1^
**Sociodemographic variables**	Men		131	55.0	38	29.0
	Women		107	45.0	23	21.5
	Age groups:	30–39 years	13	5.5	0	0
		40–49 years	29	12.2	0	0
		50–59 years	52	21.9	3	5.8
		60–69 years	47	19.8	6	12.8
		70–79 years	49	20.6	14	28.6
		≥80 years	48	20.2	38	79.2
	Retirement home residence		26	10.9	21	80.8
**Smoking**	Non-smoker		134	56.3	29	21.6
	Current smoker		22	9.2	3	13.6
	Ex-smoker		40	16.8	13	32.5
**Comorbidities**	Any comorbidity		135	56.7	55	40.7
	Hypertension		116	48.7	47	40.5
	Diabetes mellitus		52	21.9	27	51.2
	Cardiovascular disease		54	22.7	36	66.7
	Prior lung disease		50	21.0	22	44.0
	Chronic kidney failure		23	9.7	15	65.2
	Active neoplasia		8	3.4	3	37.5
	Multiple medications		77	32.4	39	50.7
	Dependence for basic activities of daily living		47	19.8	37	78.7
**Clinical findings**	Low-grade or higher fever		213	89.5	49	23.0
	Dry cough		192	80.7	52	27.1
	General malaise		151	63.5	42	27.8
	Fatigue		141	59.2	32	22.7
	Dyspnoea		146	61.3	43	29.5
	Myalgia		105	44.1	15	14.3
	Ageusia		55	23.1	1	1.8
	Anosmia		48	20.2	2	4.2
	Diarrhoea		70	29.4	8	11.4
	Productive cough		62	26.1	16	25.8
	Nausea		55	23.1	7	12.7
	Headache		48	20.2	4	8.3
	Odynophagia		23	9.7	6	26.1
	Haemoptysis		8	3.4	1	12.5
	Exanthema		3	1.3	0	0
**Total**			**238**	**100**	**61**	**25.6**

^1^ Percentage of deaths in each category of baseline variables.

Also shown in Table 1 are the proportions of deaths in the entire sample and for each category of baseline variables. About one fourth of the patients in the present series died (25.6%). No patients younger than 50 years died; as age increased, the proportion of deaths increased in an approximately exponential manner, e.g. 5.8% in patients aged 50 to 59 years (95%CI 1.2–15.9), 12.8% in those aged 60 to 69 (95%CI 4.8–25.7), 28.6% in those aged 70 to 79 (95%CI 16.6–43.3), and 79.2% in those 80 years old or more (95%CI 65.0–89.5). In our series only 5.8% (6 out of 103) of the patients with no prior comorbidities died. In contrast, 78.7% of our patients with dependence for BADL and 80.8% of those who resided in retirement homes died.

Tables 2 and 3 show the HR estimated with Cox stepwise regression models to quantify the association between hazard of death and variables recorded on admission (see Methods).

**Table 2:** Hazard ratios for the association of baseline variables with hazard of death.

Group	Variable^1^	HR^2^	95%CI	HR^3^	95%CI	HR^4^	95%CI
Demographic variables	Male	1.34	0.80–2.27				
	Age^1^	1.09	1.07–1.12	1.09	1.07–1.11	1.03	1.01–1.06
	Retirement home	1.19	0.64–2.22				
Comorbidities and medications on admission	BADL dependence	2.98	1.57–5.64	2.51	1.38–3.94		
	Hypertension	1.48	0.80–2.71				
	Diabetes mellitus	2.55	1.53–4.26	2.33	1.38–3.94	2.42	1.43–4.09
	Prior lung disease	0.93	0.52–1.64				
	Chronic kidney failure	0.97	0.51–1.83				
	Cardiovascular disease	1.93	1.08–3.46				
	Multiple medications	1.57	0.90–2.74				
Symptoms on admission	Low-grade fever	0.61	0.32–1.16				
	Cough	1.40	0.69–2.84				
	Productive cough	1.02	0.57–1.80				
	Haemoptysis	0.94	0.13–6.84				
	Dyspnoea	1.64	0.94–2.84				
	Anosmia	0.26	0.06–1.09				
	Ageusia	0.11	0.02–0.83	0.11	0.02–0.83		
	Headache	1.25	0.40–3.88				
	Nausea	0.64	0.28–1.44				
	Muscle tiredness	0.75	0.45–1.26				
	Myalgia	0.60	0.33–1.09				
	Odynophagia	1.73	0.74–4.04				
	Diarrhoea	0.57	0.27–1.22				
	General malaise	1.28	0.74–2.21				
	Gastrointestinal symptoms	0.58	0.29–1.16				
	Fatigue	0.76	0.46–1.26				
	Duration of symptoms (days)^1^	0.98	0.91–1.05				
Physical exam on admission	Fever	2.00	1.11–3.60				
	Baseline SatO_2_^1^	0.93	0.91–0.95				
	Need for O_2_	5.17	2.35–11.41				
	FiO_2_^1^	1.05	1.03–1.07				
	SatO_2_/FiO_2_^1^	0.43	0.33–0.56	0.44	0.34–0.58	0.57	0.43–0.77
X-Ray findings on admission	Opacities	2.93	1.24–6.92				
	Interstitial opacities	2.37	1.28–4.40	2.36	1.24–4.49		
	Hilar congestion	1.28	0.71–2.32				
	Pleural effusion	1.21	0.60–2.43				

^1^ Quantitative variables. Hazard ratios are expressed as increments in the hazard of death per unit increase in the variable. For dichotomous variables, the reference category was No or Absent.

^2^ Hazard ratios adjusted by age.

^3^ Hazard ratios obtained with stepwise regression models including age and the remaining variables in each group.

^4^ Hazard ratios obtained with a stepwise regression model including all variables retained in the models fitted for each group of variables.

BADL, Basic activities of daily living.

**Table 3:** Hazard ratios for the associations of each laboratory parameter with hazard of death.

Group	Variable^1^	HR^2^	95%CI	HR^3^	95%CI	HR^4^	95%CI
Laboratory values on admission	Haemoglobin	1.00	0.88–1.13				
	Lymphocytes	1.00	0.99–1.00				
	Neutrophils	1.00	0.99–1.01				
	Glycaemia	1.01	1.00–1.01	1.01	1.00–1.01		
	D-dimer	1.00	0.99–1.00				
	Total bilirubin	1.00	0.89–1.11				
	Creatinine	0.99	0.94–1.05				
	Altered kidney function^1^	1.74	1.00–3.01				
	Ferritin	1.00	1.00–1.00				
	C-reactive protein	1.00	1.00–1.00				
	Procalcitonin	1.04	1.00–1.08				
	Troponins	1.00	1.00–1.00				
	PO_2_	0.99	0.98–1.00				
	PCO_2_	1.02	0.99–1.04				
	CO_2_	1.00	1.00–1.00				
	SOFA	1.24	1.14–1.35	1.17	1.08–1.30	1.19	1.05–1.34
	CURB-65	2.44	1.85–3.21	2.53	1.81–3.55	1.73	1.23–2.43

^1^ Altered kidney function was considered as a dichotomous variable, with No as the reference category. All other variables are quantitative; hazard ratios are expressed as increments in the hazard of death per unit increase in the variable.

^2^ Hazard ratios adjusted by age.

^3^ Hazard ratios obtained with stepwise regression models including age and the remaining laboratory variables.

^4^ Hazard ratios obtained with a stepwise regression model including all variables retained in the models fitted for each group of variables (see Table 2).

SOFA, Sequential Organ Failure Assessment score; CURB-65, Score on the Confusion Urea Respiratory and Blood pressure scale.

Table 2 show the baseline variables regarding demographic data, comorbidities and medications, symptoms, physical exam and X-Ray findings on admission. The variables included in the final model as associated with higher mortality were age (3% increase in HR per 1-year increase in age; 95%CI 1–6), DM (HR 2.42; 95%CI 1.43–4.09. Elevated SatO_2_/FiO_2_ ratio was inversely related with mortality (43% decrease per 1-point increase, 95%CI 23–57). None of the other baseline variables were independently associated with mortality.

Table 3. show the laboratory parameters and prognostic scales on admission. The variables included in the final model as associated with higher mortality were SOFA score (19% increase per 1-point increase, 95%CI 5–34) and CURB-65 score (73% increase per 1-point increase, 95%CI 23–143). None of the other baseline variables were independently associated with mortality.

## Discussion

The sex and age distribution in our series of patients differs from that reported by the WHO mission in China and from data published by the Spanish National Epidemiology Centre, both of which found very similar figures [7,20]. In the present series the proportion of men was larger (55% vs. 51% in China) and median age was greater (67 years vs. 51 years in China). Our figures can be attributed in part to the greater population aging in Spain, where people aged 65 years or more account for nearly 50% of the population vs. 12% in China [21]. In addition, evolution of the pandemic in Spain has lagged behind China, and this may mean that because the patients in our series are among the first in Spain for whom these early outcomes (discharge or death) are reported, our sample comprises a high proportion of patients with factors potentially associated with more severe illness: male sex and older age [16,22,23].

Regarding the distribution of comorbidities and symptoms on hospital admission, our results are consistent with earlier studies in China [4,24,22,25], and with data from the Ministry of Health report on the status of COVID-19 in Spain as of 17 April 2020 [26]. In all series studied to date, the comorbidities associated most frequently with COVID-19 were CVD, DM and HT, and the most common clinical characteristics on admission were low-grade fever, dry cough, general malaise, fatigue, and dyspnoea. In our series, the presence of ageusia showed better prognosis of patients (HR 0.11; 95%CI 0.02–0.83) in the stepwise regression model including age and the remaining symptomatologic variables. This symptom has reported to be more frequent in younger patients and in women [27,28]. Therefore, ageusia might bias the results as older age and male sex are associated with worse prognosis. Patients with cognitive impairment also would present difficulties to detect and express this specific symptom. This hypothesis should be tested in specific studies but, in any case, the association disappeared in our final models fitted for each group of variables. Regarding smoking, although several studies have pointed a worse progression and adverse outcomes of COVID-19 [29,30], in our study we could not corroborate these results. Apart from the small number of current smokers in our sample (only 22 patients and 3 deaths among them) which may lead to high variability in the estimations for this subgroup, this fact might have two explanations. First, smoking habits were not systematically collected in all clinical records. Therefore, the existence of a considerable amount of missing data (42 patients) for this variable may have distorted the results. Second, patients with high number of comorbidities (i.e., DM, pulmonary diseases or living in retirement homes) might plausibly show a lower frequency of smoking habits given their condition, whilst their intrinsic risk of mortality is high. Again, this hypothesis should be tested in specific studies. Two findings from our crude analysis stand out: the low frequency of death in our patients with no comorbidities (5.8%), and the high percentage of deaths among patients with dependence for BADL (approaching 80%) and/or who lived in retirement homes. These three variables are closely related with each other, and with older age, as shown by the Cox regression models. Although we can venture no explanation for the reasons behind these relationships, these three variables alone or in combination appear to define a population group at high risk of death, as also suggested in earlier studies [31–36]. We believe this high risk should be taken into account in setting priorities for the indication for hospital admission. In particular, patients who live in retirement homes are likely to have most of the risk factors we identified, and in fact, it is estimated that nearly 12,200 older persons have died during the current pandemic among residents of public, joint public–private, and private retirement facilities in Spain [37]. This makes it imperative to develop specific, co-ordinated actions across all such centres during the epidemic, in order to ensure that the necessary material and human resources are available to care for these especially vulnerable patients. To date, few studies have analysed the causes of higher morbidity and mortality in this population group [38,39], and no such studies have been done in Spain. Our findings suggest the need for a specific approach to care for this group both in hospital emergency services and in residential retirement facilities.

The models we obtained with our adjusted analysis showed that in our series of patients hospitalized for COVID-19, characteristics present before SARS-CoV-2 infection that were independently associated mortality were age (exponential increase in patients 50 years old and older) and DM. A study from China based on a retrospective cohort of 191 patients identified CVD and DM as chronic diseases associated with greater mortality. However, in contrast to our finding for DM, Zhou and colleagues found that this association disappeared in their adjusted analysis [16]. In this study, associations were detected only for patient age (odds ratio 1.10 per 1-year increase in age, 95% confidence interval [95%CI] 1.03–1.17).

At least one other review of comorbidities associated with COVID-19 found that CVD and DM were among the factors most strongly associated with an increased risk of mortality, although this was based only on crude data [40].

With regard to baseline variables relating to the characteristics of SARS-CoV-2 infection on admission, those that yielded independent associations with mortality were greater need for externally supplied oxygen to maintain adequate saturation (low SatO_2_/FiO_2_ ratio as a result of ARDS) and high SOFA and CURB-65 scores. These findings are consistent with factors identified in earlier reports from countries other than Spain [7,16,41–43]. Zhou and colleagues [16] detected two risk parameters measured on admission: SOFA score (odds ratio 5.65, 95%CI 2.61–12.23) and D-dimer concentration greater than 1 μg/ml (odds ratio 18.42, 95%CI 2.64–128.55). Although D-dimer has been consistently associated with higher mortality [44,45], we could not corroborate these results in our study.

As noted in the Methods section, the main limitation of this study is the selection bias resulting from out inclusion criteria: patients who were either discharged or died, excluding those who were still hospitalized at the end of the recruitment period. Nonetheless, we feel the use of Cox regression models minimized the impact of this bias on the magnitude of our estimated associations, given that the estimates were a function of time to death. Because our patients comprise the first sample of people admitted with the characteristics reported here, the duration of follow-up was brief and this may have led to the selection of “extreme” cases, i.e. those who were the first to be discharged or the first to die in our hospital. This factor makes our findings especially useful for studies of the first patients to be hospitalized in other settings. These considerations, along with the chronology of the COVID-19 pandemic in Spain, make it likely that the patients selected for this study differed from those who were still hospitalized at the end of the recruitment period. In other words, compared to subsequently admitted patients, those analysed here may have had more and/or more severe clinical signs and symptoms as grounds for admission. Another potential source of error is the lack of adjustment for variables we did not measure, either because they were not recorded in the medical records or were not considered relevant in light of our current (incomplete, constantly changing) knowledge of COVID-19. There is another potential bias concerning the selection of patients which are mainly elderly patients coming from a localized region in Spain. Therefore, the results could not be widely generalized. A final consideration is that we designed a monocentric study with a limited number of patients, while a great number of different factors were analysed as independent variables. This limited sample size necessitated a sequential process to develop our models, with a stepwise approach and low number of predictive variables.

## Conclusions

In addition to the close association between mortality and comorbidities, dependence for BADL and living in a retirement facility in our crude analysis, the main baseline factors that were independently associated in our adjusted analysis with mortality in patients hospitalized for COVID-19 were older age, DM, low SatO_2_/FiO_2_ ratio, and high SOFA and CURB-65 scores. These characteristics define a profile of patients in Spain with an elevated mortality rate for whom a specific approach to clinical management is urgently needed.
